# Is Gestational Hypertension Protective against Perinatal Mortality in Twin Pregnancies?

**DOI:** 10.1371/journal.pone.0094865

**Published:** 2014-04-14

**Authors:** Qi-Guang Luo, Ji-Yan Zhang, Wei-Wei Cheng, Francois Audibert, Zhong-Cheng Luo

**Affiliations:** 1 Department of Obstetrics and Gynecology, Sainte-Justine Hospital, University of Montreal, Montreal, Canada; 2 Department of Obstetrics and Gynecology, International Peace Maternity and Child Health Hospital, Shanghai Jiao-Tong University School of Medicine, Shanghai, China; 3 Department of Statistics and Actuarial Science, University of Waterloo, Waterloo, Canada; 4 Ministry of Education-Shanghai Key Laboratory of Children’s Environmental Health, Xinhua Hospital, Shanghai Jiao-Tong University School of Medicine, Shanghai, China; Brown University, United States of America

## Abstract

**Background:**

Pregnancy-induced or gestational hypertension is a common pregnancy complication. Paradoxically, gestational hypertension has been associated with a protective effect against perinatal mortality in twin pregnancies in analytic models (logistic regression) without accounting for survival time. Whether this effect is real remains uncertain. This study aimed to validate the impact of gestational hypertension on perinatal mortality in twin pregnancies using a survival analysis approach.

**Methods:**

This was a retrospective cohort study of 278,821 twin pregnancies, using the U.S. 1995–2000 matched multiple birth dataset (the largest dataset available for multiple births). Cox proportional hazard models were applied to estimate the adjusted hazard ratios (aHR) of perinatal death (stillbirth and neonatal death) comparing gestational hypertensive vs. non-hypertensive pregnancies controlling for maternal characteristics and twin cluster-level dependence.

**Results:**

Comparing births in gestational hypertensive vs. non-hypertensive twin pregnancies, perinatal mortality rates were significantly lower (1.20% vs. 3.38%), so were neonatal mortality (0.72% vs. 2.30%) and stillbirth (0.48% vs. 1.10%) rates. The aHRs (95% confidence intervals) were 0.34 (0.31–0.38) for perinatal death, 0.31 (0.27–0.34) for neonatal death, and 0.45 (0.38–0.53) for stillbirth, respectively. The protective effect of gestational hypertension against perinatal death became weaker over advancing gestational age; the aHRs in very preterm (<32 weeks), mild preterm (32–36 weeks) and term (37+ weeks) births were 0.29, 0.48 and 0.76, respectively. The largest risk reductions in neonatal mortality were observed for infections and immaturity-related conditions.

**Conclusions:**

Gestational hypertension appears to be beneficial for fetal survival in twin pregnancies, especially in those ending more prematurely or for deaths due to infections and immaturity-related conditions. Prospective studies are required to rule out the possibility of unmeasured confounders.

## Introduction

Pregnancy-induced hypertension or gestational hypertension is a common pregnancy complication affecting 5% to 10% of all pregnancies [1]. It occurs more frequently in multiple pregnancies most of which (about 97%–98%) are twin pregnancies [2], [3]. Multiple pregnancies have become increasingly common in recent decades [4], but few studies have addressed the potential differential needs in the management of pregnancy-specific complications in multiple vs. singleton pregnancies. In the presence of multiple fetuses, the maternal/placental burdens to support more fetuses create a substantial physiological difference between multiple vs. singleton pregnancies that may explain the elevated risks of some gestational-specific complications in multiple pregnancies. In case of gestational hypertension, the maternal elevation in blood pressure may be considered a developmental competing survival mechanism to increase blood/nutrients supply to the fetus, but this is a delicate risks/benefits balance because high blood pressure itself poses a risk to the mother and fetus [5]. This developmental need for higher blood pressure to increase fetal blood/nutrients supply would be likely stronger in the presence of multiple fetuses. Therefore, it is a plausible hypothesis that the development of gestational hypertension may be beneficial to fetal survival in multiple pregnancies with a much greater need for blood/nutrients supply. Indeed, gestational hypertension has been associated with a reduced risk of neonatal death in twin but not singleton pregnancies [6]. A reduced risk of fetal and infant mortality has been reported in gestational hypertensive vs. non-hypertensive twin pregnancies ending in preterm births [7]. In contrast, gestational hypertension has been generally associated with adverse birth outcomes in singleton pregnancies [6], [8]. If this paradoxically beneficial effect of gestational hypertension on fetal survival in multiple pregnancies is real, there may be a need for differential clinical management recommendations in multiple vs. singleton gestational hypertensive pregnancies. That is, the optimal policy for the management of gestational hypertension in singleton pregnancies may be not the optimal choice in multiple pregnancies. However, previous studies used logistic regression to model the risk of perinatal mortality without accounting for the duration of fetal survival [6], [7]. In estimating the risk of perinatal mortality, survival analysis accounting for the duration of survival would be a more efficient and appropriate model. This study aimed to validate the impact of gestational hypertension on the risk of perinatal mortality in twin pregnancies using a survival analysis approach.

## Methods

This was a retrospective cohort study using the U.S. matched multiple birth dataset *1995–2000* provided by the National Center for Health Statistics (NCHS) [9]. It is the largest linked dataset available for multiple births. Subjects included in the present study must meet the following inclusion criteria: 1) births in twin pregnancies with non-missing value for gestational hypertension; 2) pregnancies without chronic hypertension; 3) gestational age at delivery between 20 and 42 weeks inclusive (twin births outside of this gestational age range are extremely rare, and likely recording/transcription errors). In cases one twin had valid data but the other twin did not or was missing in a twin set, the twin with valid data remained in the analysis cohort. Therefore, the total number of births in the final study cohort is slightly less than 2 times the total number of pregnancies. We did not exclude births with missing data on birth weight (n = 8615) (1.6% of all births) because a significant proportion of perinatal deaths (7187 of 17779) (40.4%) had missing data on birth weight. The main interest is the risk of perinatal death which could be estimated irrespective of missing data on birth weight. Birth weight was set to missing for births at very extreme birth weights (<500 g, or >6000 g) (probably recording or transcription errors). Births with missing data on birth weight were allowed to drop out in birth weight-specific analyses. A total of 278,821 twin pregnancies (555,457 twin births) constituted the final study cohort. Research ethics approval was waived by the Sainte-Justine hospital research ethics board because the study was based on the anonymized matched multiple birth dataset downloadable from the NCHS website.

During the study period in the U.S. 1995–2000, the diagnosis of pregnancy-induced or gestational hypertension was according to the then commonly accepted criteria: two or more occasions in blood diastolic pressure ≥90 mmHg or systolic pressure ≥140 mmHg, taken ≥4 hours apart and occurring after 20 weeks of gestation without proteinuria [10]. In the NCHS multiple birth database, it is impossible to distinguish between gestational hypertension (without proteinuria) and preeclampsia (gestational hypertension with proteinuria).

The primary exposure of interest was gestational hypertension. Other study variables included fetal sex, gestational age (weeks), birth weight (grams), mode of delivery (caesarean, vaginal), maternal race (white, black, others), marital status (not married, married), age (<20, 20–34, 35+ years), education [<12, 12 (high school graduation), 13–15 (some post-secondary), and 16+ years (college or higher)], parity (primiparous: yes/no), maternal smoking (yes/no), and the reported presence of any other major maternal illnesses (yes/no) including diabetes, heart disease, genital herpes, renal disease, acute or chronic lung disease and Rh sentization. The number of missing values was <2% for most study variables, except for smoking (17.8% missing), mode of delivery (36.4% missing), and the presence of major maternal illnesses (7.4% missing).

The primary outcome was perinatal death including stillbirth (fetal deaths at 20 or more weeks gestation) and neonatal death (deaths during the first 4 weeks of postnatal life). We also examined cause-specific neonatal mortality (congenital anomalies, asphyxia, immaturity related conditions, infections, sudden infant death syndrome, others) according to the International Collaborative Effort (ICE) on Perinatal and Infant Mortality [11]. Causes of death are missing for all stillbirths in the NCHS birth data.

The data analysis unit was the mother for maternal/pregnancy variables (e.g. gestational hypertension), but the fetus/newborn for perinatal outcomes (perinatal death, stillbirth, neonatal death). Marginal Cox regression models for clustered data [12] were applied to estimate the crude and adjusted hazard ratios (aHR) and 95% confidence interval (CI) of perinatal death, stillbirth and neonatal death comparing gestational hypertensive vs. non-hypertensive pregnancies accounting for twin set-cluster level dependence. The survival time (time to event or outcome) variable in the Cox models was gestational age (in weeks) for stillbirth, and gestational age at delivery plus the number of postnatal surviving weeks for neonatal deaths, and gestational age at delivery plus 4 weeks for all surviving babies (right-censored). The aHR were adjusted for maternal race, age, marital status, parity, smoking, other reported maternal illnesses, fetal sex and mode of delivery.

To gain insight into clinical characteristics of risk changes, the HRs of perinatal death were examined by important clinical characteristics: mode of delivery (caesarean, vaginal), gestational age (very preterm <31 weeks, mild preterm 32–36 weeks, term ≥37 weeks), birth weight (very low <1500 g, low 1500–2499 g, normal ≥2500 g), and fetal growth – small, appropriate or large for gestational age (SGA <10^th^ percentile, AGA 10–90^th^ percentiles, LGA >90^th^ percentile) according to sex- and gestational age-specific birth weight percentiles for non-malformation births to mothers without smoking and without any reported major maternal illness in the study cohort.

Births with missing data on co-variable were allowed to drop out in all multivariate adjustment models, except for smoking and mode of delivery for which the proportion of missing was high (17.8% for smoking, 36.4% for mode of delivery); the missing was included as a valid category to avoid large numbers of drop-outs in the adjusted risk models. For gestational age specific analysis, fetus-at-risk approach was applied to avoid a potential “collider” effect of stratification by gestational age at birth [13]. The fetuses-at-risk denominator is the number of all fetuses at risk of death (both born and yet unborn babies). For example, in calculating perinatal mortality for births at 32–36 weeks gestation, those yet unborn babies (births at >36 weeks) were considered at risk and included in the denominator. All data management and analyses were carried out using Statistical Analysis System, version 9.2 (SAS Institute, Cary, North Carolina) [14]. Two-tailed p values <0.05 were considered statistically significant.

## Results

Gestational hypertension was reported in 22,839 out of 278,821 twin pregnancies (8.2%). There were significant differences in maternal and pregnancy characteristics comparing gestational hypertensive vs. non-hypertensive pregnancies **(** **Table 1** **)**. Mothers with gestational hypertension were more likely to be white (80.5% vs. 78.8%), primiparous (59.6% vs. 39.7%), over the age of 35 years (20.5% vs. 18.6%), to have college or higher education (33.0% vs. 30.0%), to have other major maternal illnesses (11.1% vs. 6.3%), to deliver a preterm (65.0% vs. 55.4%) or SGA baby (12.0% vs. 10.2%) or have a caesarean delivery (61.8% vs. 51.2%), but less likely to be a smoker (7.5% vs. 10.8%), unmarried (26.0% vs. 27.6%), or to deliver a LGA baby (9.0% vs. 9.6%). There was no significant difference in the proportion of births with reported congenital anomalies overall (2.2% vs. 2.2%, p = 0.52). Compared gestational hypertensive vs. non-hypertensive twin pregnancies ending in preterm deliveries, mothers were more likely to have reported other major illnesses (11.7% vs. 6.7%), infants were more likely to be SGA (11.1% vs. 9.5%), but less likely to have congenital anomalies (2.5% vs. 3.1%).

**Table 1:** Maternal, pregnancy and newborn characteristics in gestational hypertensive versus non-hypertensive twin pregnancies in the study population, U.S. 1995–2000.

	Gestationalhypertensive	Non-hypertensive	
	pregnancy	pregnancy	P
Number of twin pregnancies	22839	255982	
Number of included twin births	45272	510185	
***Mothers,*** * n (%)*			
Race			<0.0001
Black	3632 (15.9)	44190 (17.3)	
White	18385 (80.5)	201628 (78.8)	
Others	812 (3.6)	10164 (4.0)	
Unmarried	5921 (26.0)	70461 (27.6)	<0.0001
Age (years)			<0.0001
<20	1815 (7.9)	17738 (6.9)	
20–34	16340 (71.6)	190704 (74.5)	
≥35	4674 (20.5)	47540 (18.6)	
Education			<0.0001
Lower than high school	2944 (13.0)	40498 (16.0)	
High School (12y)	6677 (29.6)	77421 (30.6)	
Some college (13–15y)	5519 (24.4)	58318 (23.1)	
College or higher (≥16 y)	7450 (33.0)	76563 (30.3)	
*Pregnancy, n (%)*			
Primiparous	13579 (59.6)	101534 (39.7)	<0.0001
Maternal smoking^t^	1436 (7.5)	22708 (10.8)	<0.0001
Other maternal major illness^c^	2338 (11.1)	15028 (6.3)	<0.0001
Caesarean delivery^t^	8744 (61.8)	83486 (51.2)	<0.0001
***Newborns,*** * n (%)*			
Preterm birth (<37 weeks)	29405 (65.0)	282759 (55.4)	<0.0001
Low birth weight (<2500 g)	26503 (58.9)	268264 (53.5)	<0.0001
SGA (<10^th^ percentile)*	5376 (12.0)	51042 (10.2)	<0.0001
LGA (>90^th^ percentile)*	4027 (9.0)	48175 (9.6)	<0.0001
Congenital anomalies	1022 (2.3)	11883 (2.4)	0.36
***Among preterm deliveries,*** * n (%)*			
Other maternal major illness^c^	1580 (11.7)	8781 (6.7)	<0.0001
SGA (<10^th^ percentile)*	3226 (11.1)	26224 (9.5)	<0.0001
LGA (>90^th^ percentile)*	2781 (9.6)	27030 (9.8)	0.12
Congenital anomalies	725 (2.5)	8533 (3.1)	<0.0001

Data presented are n (%). P values are from Chi-square tests for differences between diabetic and non-diabetic pregnancies. *SGA = Small-for-gestational-age <10^th^ percentile, LGA = large-for-gestational-age >90^th^ percentile, according to birth weight percentiles in non-malformation births to non-smoking mothers in the study cohort.

c One or more of the following conditions: diabetes, heart disease, acute or chronic lung disease, renal disease, genital herpes and RH sensitization.

t There were significant numbers of missing value (>10%) for smoking (n = 49494 mothers) (17.8%) and mode of delivery (101368 mothers) (36.4%). The numbers of missing for other variables were: race 0, marital status 705 (0.3%) mothers, age 0, education 3389 (1.2%) mothers, parity 12 (0.0%), other maternal illness 20721 (7.4%) mothers, preterm birth 0, low birth weight, SGA or LGA 8615 (1.5%) newborns. The rates for smoking and caesarean section, SGA, et al. are based on births with non-missing information.

**Figure 1:**
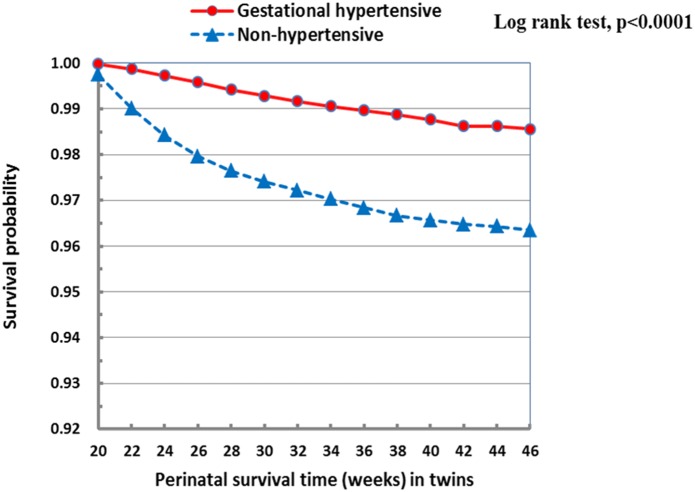
Survival probabilities during the perinatal period (from 20 weeks gestation to 4 weeks postpartum) in gestational hypertensive vs. non-hypertensive twin pregnancies.

There were a total of 17,779 perinatal deaths (3.20%) in the study cohort. Survival probabilities during the perinatal period (from 20 weeks gestation to 4 weeks postpartum) were significantly higher in gestational hypertensive vs. non-hypertensive twin pregnancies **(** **Figure 1** **)**. Compared gestational hypertensive vs. non-hypertensive pregnancies, perinatal, neonatal mortality and stillbirth rates were all significantly lower (1.20% vs. 3.38%, 0.72% vs. 2.30% and 0.48% vs. 1.10%, respectively), so were the mortality hazards. The aHRs (95% CIs) were 0.34 (0.31–0.38) for perinatal death, 0.31 (0.27–0.34) for neonatal death, and 0.45 (0.38–0.53) for stillbirth, respectively **(** **Tables 2** **,** **3** **,** **4** **)**.

**Table 2:** Perinatal mortality in gestational hypertensive versus non-hypertensive twin pregnancies, U.S. matched multiple birth data 1995–2000.

	Perinatal	mortality (rate)		
	GestationalHypertensive	Non-hypertensive	Crude	Adjusted*
	Pregnancy	Pregnancy	HR (95% CI)	HR (95% CI)
	n/total (%)	n/total (%)		
All births (n = 555457)	544/45272 (1.20)	17235/510185 (3.38)	**0.35 (0.32–0.38)**	**0.34 (0.31–0.38)**
Mode of delivery				
Caesarean section	176/17746 (0.99)	3243/173111 (1.87)	**0.53 (0.45–0.61)**	**0.55 (0.47–0.65)**
Vaginal	111/10318 (1.08)	6409/151422 (4.23)	**0.25 (0.21–0.30)**	**0.20 (0.16–0.25)**
Unknown	257/17208 (1.49)	7583/185652 (4.08)	**0.36 (0.32–0.41)**	**0.34 (0.30–0.39)**
Gestational age^t^				
Very preterm (≤31 weeks)	361/45272 (0.80)	13834/510185 (2.71)	**0.29 (0.26–0.32)**	**0.29 (0.25–0.32)**
Mild preterm (32–36 weeks)	106/44374 (0.24)	2241/482008 (0.46)	**0.51 (0.42–0.62)**	**0.48 (0.38–0.60)**
Term (≥37 weeks)	77/39592 (0.19)	1160/427177 (0.27)	**0.78 (0.62–0.98)**	**0.76 (0.58–0.98)**
Birth weight**				
Very low (<1500 g)	230/3268 (7.04)	7957/48779 (16.31)	**0.38 (0.33–0.43)**	**0.42 (0.37–0.49)**
Low (1500–2499 g)	93/23235 (0.40)	1686/219485 (0.77)	**0.52 (0.43–0.65)**	**0.52 (0.41–0.66)**
Normal (≥2500 g)	37/18465 (0.20)	589/233610 (0.25)	0.83 (0.59–1.15)	0.75 (0.51–1.09**)**
Fetal growth**				
SGA (<10^th^)	129/5376 (2.40)	2434/51042 (4.77)	**0.51 (0.42–0.60)**	**0.53 (0.43–0.64)**
AGA (10^th^–90^th^)	201/35565 (0.57)	7283/402656 (1.81)	**0.31 (0.69–0.36)**	**0.29 (0.25–0.34)**
LGA (>90^th^)	30/4027 (0.74)	515/48175 (1.07)	0.70 (0.49–1.01)	**0.57 (0.37–0.88)**

HR = Hazard ratio; CI =  confidence interval.

*Hazard ratios adjusted for maternal race, marital status, age, education, parity, smoking, other maternal major illnesses, fetal sex, mode of delivery and twin-cluster level dependence in Cox regression models.

**There were a significant number of perinatal deaths with missing birth weights.

t Gestational age group-specific mortality rates and hazard ratios were calculated using the number of foetuses at risk and the number of perinatal deaths in the time interval specified.

**Table 3:** Neonatal mortality in gestational hypertensive versus non-hypertensive twin pregnancies, U.S. matched multiple birth data 1995–2000.

	Neonatal	mortality (rate)^a^		
	GestationalHypertensive	Non-hypertensive	Crude	Adjusted*
	Pregnancy	Pregnancy	HR (95% CI)	HR (95% CI)
	n/total (%)	n/total (%)		
All live births (n = 555182)	326/45054 (0.72)	11599/504549 (2.30)	**0.31 (0.28–0.35)**	**0.31 (0.27–0.34)**
Mode of delivery				
Caesarean section	139/17709 (0.78)	2703/172571 (1.57)	**0.500 (0.42–0.59)**	**0.51 (0.43–0.62)**
Vaginal	73/10280 (0.71)	4936/149949 (3.29)	**0.21 (0.17–0.27)**	**0.18 (0.14–0.23)**
Unknown	114/17065 (0.67)	3960/182029 (2.18)	**0.31 (0.25–0.37)**	**0.29 (0.24–0.35)**
Gestational age^t^				
Very preterm (≤31 wks)	233/45054 (0.52)	9930/504549 (1.97)	**0.26 (0.23–0.30)**	**0.26 (0.23–0.30)**
Mild preterm (32–36 wks)	48/44284 (0.11)	1081/480276 (0.23)	**0.48 (0.36–0.64)**	**0.47 (0.34–0.63)**
Term (≥37 wks)	77/39592 (0.19)	1160/427177 (0.27)	**0.78 (0.62–0.98)**	**0.76 (0.58–0.98)**
Birth weight**				
Very low (<1500 g)	146/3184 (4.59)	6143/46965 (13.08)	**0.32 (0.27–0.37)**	**0.36 (0.31–0.43)**
Low (1500–2499 g)	48/23190 (0.21)	893/218692 (0.41)	**0.51 (0.38–0.69)**	**0.49 (0.36–0.67)**
Normal (≥2500 g)	25/18453 (0.14)	323/233344 (0.14)	1.04 (0.69–1.56)	0.95 (0.61–1.48)
Fetal growth**				
SGA (<10^th^)	64/5311 (1.21)	1463/50071 (2. 92)	**0.42 (0.33–0.54)**	**0.42 (0.32–0.55)**
AGA (10^th^–90^th^)	139/33503 (0.39)	5574/400948 (1.39)	**0.28 (0.24–0.33)**	**0.26 (0.22–0.32)**
LGA (>90^th^)	16/4013 (0.40)	322/47982 (0.67)	**0.60 (0.36–0.99)**	**0.58 (0.34–0.97)**

HR = Hazard ratio; CI =  confidence interval.

*Hazard ratios adjusted for maternal race, marital status, age, education, parity, smoking, other maternal major illnesses, fetal sex, mode of delivery and twin-cluster level dependence in Cox regression models.

**There were a significant number of neonatal deaths with missing birth weights.

a The denominators are smaller than those numbers in Table 2 or 4 because neonatal mortality was calculated based on live births only as the denominators, while perinatal mortality and stillbirth rates were calculated based on all births (live births plus stillbirths) as the denominators.

t Gestational age group-specific mortality rates and hazard ratios were calculated using the number of foetuses at risk and the number of neonatal deaths in the time interval specified.

**Table 4:** Stillbirth in gestational hypertensive versus non-hypertensive twin pregnancies, U.S. matched multiple birth data 1995–2000.

	Stillbirth	(rate)		
	Gestational Hypertensive	Non-hypertensive	Crude	Adjusted*
	Pregnancy	Pregnancy	HR (95% CI)	HR (95% CI)
	n/total (%)	n/total (%)		
All births (n = 561157)	218/45272 (0.48)	5636/510185 (1.10)	**0.44 (0.39–0.51)**	**0.45 (0.38–0.53)**
Mode of delivery				
Caesarean section	37/17746 (0.21)	540/173111 (0.31)	**0.71 (0.51–0.99)**	0.84 (0.58–1.21)
Vaginal	38/10318 (0.37)	1473/151422 (0.97)	**0.38 (0.27–0.52)**	**0.27 (0.18–0.42)**
Unknown	143/17208 (0.83)	3623/185652 (1.95)	**0.44 (0.37–0.51)**	**0.44 (0.36–0.54)**
Gestational age^t^				
Very preterm (≤31 wks)	128/45272 (0.28)	3904/510185 (0.77)	**0.36 (0.31–0.43)**	**0.37 (0.30–0.46)**
Mild preterm (32–36 wks)	58/41622 (0.14)	1160/447296 (0.26)	**0.56 (0.43–0.73)**	**0.51 (0.36–0.70)**
Term (≥37 wks)	32/15867 (0.20)	572/227426 (0.25)	0.84 (0.61–1.25)	0.93 (0.62–1.38)
Birth weight**				
Very low (<1500 g)	84/3268 (2.57)	1814/48779 (3.72)	**0.54 (0.43–0.67)**	**0.61 (0.47–0.80)**
Low (1500–2499 g)	45/23235 (0.19)	793/219485 (0.36)	**0.58 (0.43–0.78)**	**0.61 (0.43–0.88)**
Normal (≥2500 g)	12/18465 (0.06)	266/233610 (0.11)	0.67 (0.38–1.20)	0.56 (0.27–1.15)
Fetal growth**				
SGA (<10^th^)	65/5376 (1.21)	971/51042 (1.90)	**0.69 (0.54–0.88)**	**0.76 (0.57–1.02)**
AGA (10^th^–90^th^)	62/35565 (0.17)	1709/402657 (0.42)	**0.42 (0.32–0.54)**	**0.41 (0.30–0.56)**
LGA (>90^th^)	14/4027 (0.35)	193/48175 (0.40)	0.92 (0.53–1.58)	0.59 (0.27–1.30)

HR = Hazard ratio; CI =  confidence interval.

*Hazard ratios adjusted for maternal race, marital status, age, education, parity, smoking, other maternal major illnesses, fetal sex, mode of delivery and twin-cluster level dependence in Cox regression models.

**There were a significant number of stillbirths with missing birth weights.

t Gestational age group-specific mortality rates and hazard ratios were calculated using the number of foetuses at risk and the number of stillbirths in the time interval specified.

Stratified analyses by gestational age revealed that the protective effect of gestational hypertension against perinatal death diminished as pregnancy approached term. The aHR of perinatal death comparing gestational hypertensive vs. non-hypertensive pregnancies was 0.29 for very preterm births, 0.48 for mild preterm births, and 0.76 for term births, respectively (all p<0.001) **(** **Table 2** **)**. Similarly, the aHR for neonatal death was 0.26 for very preterm births, 0.47 for mild preterm births, and 0.76 for term births, respectively **(** **Tables 3** **)**. For stillbirth, the risk difference became non-significant for term births in hypertensive vs. non-hypertensive pregnancies **(** **Table 4**). Stratified analyses by birth weight or birth weight for gestational age also showed a tapering protective effect of gestational hypertension against perinatal death with increasing birth weight; low birth weight, SGA and AGA births experienced a significant protective effect, while the risk difference became non-significant for newborns reaching normal birth weight **(** **Table 2**). Stratified analyses by mode of delivery indicated that the protective effect was stronger for vaginal births (aHR = 0.20) than for caesarean births (aHR = 0.55).

Cause-specific neonatal mortality analyses showed that gestational hypertension was associated with a significantly lower risk of neonatal death due to any cause (congenital anomalies, immaturity related conditions, asphyxia, infections, other causes) other than sudden infant death syndrome **(** **Table 5** **)**. The strongest protective effects were observed for death due to infections (aHR = 0.23) and immaturity-related conditions (aHR = 0.26).

**Table 5:** Cause-specific neonatal mortality in gestational hypertensive versus non-hypertensive twin pregnancies, U.S. matched multiple birth data 1995–2000.

	Cause-specific	neonatal mortality^a^		
	GestationalHypertensive	Non-hypertensive	Crude	Adjusted*
	Pregnancy	Pregnancy	HR (95% CI)	HR (95% CI)
	n (%)	n (%)		
Live births, n	45054	504549		
Congenital anomalies	54 (0.12)	1426 (0.28)	**0.40 (0.30–0.54)**	**0.39 (0.29–0.52)**
Asphyxia	25 (0.06)	831 (0.16)	**0.35 (0.23–0.52)**	**0.32 (0.21–0.48)**
Immaturity-related	145 (0.32)	6281 (1.24)	**0.27 (0.23–0.32)**	**0.26 (0.22–0.31)**
Infections	41 (0.09)	1749 (0.35)	**0.25 (0.18–0.35)**	**0.23 (0.16–0.33)**
SIDS	6 (0.013)	79 (0.016)	0.81 (0.33–2.00)	0.82 (0.32–2.10)
Others	55 (0.12)	1233 (0.24)	**0.54 (0.41–0.72)**	**0.52 (0.39–0.69)**

HR = Hazard ratio; CI =  confidence interval; SIDS =  sudden infant death syndrome.

*Hazard ratios adjusted for maternal race, marital status, age, education, parity, smoking, other maternal major illnesses, fetal sex, mode of delivery and twin-cluster level dependence in Cox regression models.

a The denominators are smaller than those numbers in Table 2 or 4 because neonatal mortality was calculated based on live births only as the denominators, while perinatal mortality and stillbirth rates were calculated based on all births (live births plus stillbirths) as the denominators.

Among the 45,272 births in gestational hypertensive pregnancies, 2945 births (6.5%) were in pregnancies complicated by both gestational hypertension and diabetes. The results were very similar if these 2945 births were excluded from the analyses. If births with any reported congenital anomalies (n = 12,905) were excluded from the analyses, similar protect effects were observed for gestational hypertension against perinatal death, stillbirth and neonatal death. Compared gestational hypertensive vs. non-hypertensive twin pregnancies, the aHR (95% CI) were 0.33 (0.30–0.37) for perinatal death, 0.44 (0.37–0.57) for stillbirth, and 0.29 (0.25–0.33) for neonatal death, respectively.

## Discussion

### Main Findings

To our knowledge, this is the first report on the association of gestational hypertension with perinatal mortality in twin pregnancies using a survival analysis approach. The results suggest a protective effect of gestational hypertension against perinatal death in twin pregnancies. This protective effect appears to diminish as pregnancy advances towards term, and become non-significant for newborns reaching normal birth weight. Also, we first observed that the strongest risk reductions in neonatal mortality comparing gestational hypertensive vs. non-hypertensive pregnancies were for deaths due to immaturity-related conditions and infections.

### Comparisons with Findings in Previous Studies

Although gestational hypertension is generally associated with adverse neonatal outcomes in singletons [6], [8], the same condition has been associated with a beneficial impact on the survival of newborns in twin pregnancies [6], [7]. The current study has overcome a major limitation in previous studies based on logistic regression to model the mortality risk ignoring a crucial piece of information - survival time. In time-dependent circumstances, Cox proportional hazards models are more efficient than logistic regression models in ascertaining the true risk differences [15]. A previous study reported that the risk reductions in stillbirth and neonatal death may be limited to preterm twins comparing gestational hypertensive vs. non-hypertensive pregnancies [7]. In contrast, we observed moderate and statistically significant risk reductions in overall perinatal mortality and neonatal morality in both term and preterm twins.

The protective effect of gestational hypertension against perinatal death in twins seems to be counterintuitive, since hypertension is a known risk factor of adverse perinatal outcomes in singletons [6], [8]. The reasons and mechanisms underlying the lower risk of perinatal death in gestational hypertensive vs. non-hypertensive twin pregnancies are unclear. One may speculate that non-hypertensive twin pregnancies may experience a higher perinatal mortality because they may be more frequently complicated by other serious maternal illnesses or birth defects than gestational hypertensive twin pregnancies. However, this is not the case. To the contrary, we observed that other maternal illnesses were more frequent in gestational hypertensive vs. non-hypertensive pregnancies, while reported birth defects were similarly frequent **(** **Table 1**). Alternatively, one may speculate that twin fetuses may benefit from better blood/nutrients supply to promote better fetal growth in gestational hypertensive pregnancies. This appears not to be the case too. We observed a higher rate of SGA birth in gestational hypertensive vs. non-hypertensive pregnancies. Perinatal mortality rate was lower despite higher rates of preterm and SGA births in gestational hypertensive pregnancies, suggesting that higher blood pressure may carry some benefits to twin fetuses other than promoting fetal growth. Interestingly, cause-specific mortality risk analyses revealed that the strongest protective effects of gestational hypertension against neonatal death were for deaths due to immaturity-related conditions and infections. This raises the possibility that higher blood pressure may promote fetal maturity and immune functional development. However, this interpretation is purely speculative. Another possibility is that anti-hypertensive medications may be somewhat beneficial for fetal survival. Indeed, it has long been suspected that labetalol, a common drug in the treatment of gestational hypertension, may promote fetal lung maturation to lower perinatal mortality [16]. Of various anti-hypertensive drugs, it remains uncertain which agent is the best in terms of risks/benefits in the treatment of hypertension in pregnancy [17]. More research data are needed on the impact of anti-hypertensive agents on perinatal mortality.

The diminishing benefit of gestational hypertension against perinatal death with advancing gestational age in twin pregnancies could be a consequence of persistent high blood pressure, because high blood pressure may strain the placenta over time. Unfortunately, we had no data on the timing of onset and duration of gestational hypertension and blood pressure levels. Irrespective of gestational hypertension, pregnancies ending in preterm births are unhealthy by definition compared to those ending in term births. Alternatively, one may speculate that non-hypertensive pregnancies ending in preterm births may be even sicker than hypertensive pregnancies ending in preterm births, resulting in a stronger observed protective effect of gestational hypertension against perinatal death in preterm twins. However, even among preterm births, we observed a higher rate of other serious maternal illnesses in gestational hypertensive vs. non-hypertensive twin pregnancies. There is a possibility that other unreported serious maternal illnesses or fetal problems might have been more common in non-hypertensive pregnancies ending in preterm births. Large prospective cohort studies are required to clarify this question and rule out the possibility of unmeasured confounders.

The observed protective effect of gestational hypertension against perinatal mortality is similar to the recently reported protective effect of diabetes in pregnancy (mostly, gestational diabetes) against perinatal mortality in twin pregnancies [18]. We speculate that gestational-specific elevations in both blood pressure and glucose may represent a competing developmental survival mechanism for fetuses to extract more nutrients from the mother that may confer a perinatal survival benefit in multiple pregnancies.

### Limitations

The NCHS birth database could not allow a distinction between gestational hypertensive (without proteinuria) and preeclampsia (with proteinuria). We had no data on the severity of gestational hypertension and other associated adverse conditions. Severe hypertension or preeclampsia is a more serious condition that is associated with adverse outcomes such as placental abruption and stillbirth [19]. It is unknown but likely that the protective effect of gestational hypertension against perinatal death in twins may be restricted to mild and well-controlled hypertensive pregnancies. This question may be answered in large prospective cohort studies with data to distinguish gestational hypertension from preeclampsia.
